# Experience and Outcomes of a Dedicated Cardio-Nephrology Service: A Step toward Integrated Cardiovascular-Kidney-Metabolic Syndrome Care

**DOI:** 10.34067/KID.0000001096

**Published:** 2025-12-24

**Authors:** Karen de Wolski, David K. Prince, David Mariuma, Nayan Arora, Tejas N. Velu, Ayushi Gupta, Nisha Bansal

**Affiliations:** 1 Division of Nephrology, Department of Medicine, University of Washington, Seattle, Washington; 2 Division of Nephrology and Hypertension, Department of Medicine, Stony Brook University, Stony Brook, New York; 3 Division of Nephrology and Hypertension, Department of Medicine, Oregon Health Sciences University, Portland, Oregon; 4 Department of Medicine, University of Washington, Seattle, Washington

**Keywords:** dialysis, diuretics, cardiovascular-kidney-metabolic (CKM) syndrome, cardiorenal syndrome

## Abstract

**Key Points:**

- Patients hospitalized with concomitant cardiac and kidney disease have high morbidity. Interdisciplinary care models may help improve outcomes.
- This descriptive single-center study shows the outcomes and feasibility of a cardio-nephrology service to care for patients with cardiovascular-kidney-metabolic syndrome.

**Background:**

The cardiovascular-kidney-metabolic syndrome, estimated to affect up to 90% of US adults, is increasingly recognized as a disease spectrum that requires an interdisciplinary approach. The purpose of this descriptive study was to characterize the patients and the clinical outcomes of a specialized cardio-nephrology inpatient service. These data were compared with data from comparable patients before the service's launch.

**Methods:**

This was a retrospective observational study of a specialized Kidney-Heart service that was launched at the University of Washington in 2020 to serve as the nephrology service for patients hospitalized with primary cardiac disease. Chart review was pursued to obtain patient demographics, reason for consult and hospital outcomes in the first 2.5 years of the service. We also obtained data from a historical cohort of patients with a cardiology diagnosis seen by the general nephrology consult service as a comparator. Descriptive analyses were performed to characterize demographics, consult categories, and primary reasons for hospitalization in patients seen on the Kidney-Heart service versus the historical cohort.

**Results:**

The mean age for the patients seen on the Kidney-Heart service was 63 (SD 15) versus 60 (SD 15) years in the comparator cohort (*P* < 0.001). For the Kidney-Heart service, AKI was the most common reason for consult (57.7%), followed by CKD G5D (30.9%), diuretic management (8.9%), and electrolyte abnormalities (7.8%). Patients seen by the Kidney-Heart service were most commonly hospitalized for decompensated heart failure and cardiogenic shock (46.9%). The use of mechanical circulatory support was common (22%), and 48.2% of those patients required dialysis. AKI dialysis (36.7 versus 42%, *P* = 0.05) and mortality rates (16.5% versus 25%, *P* < 0.01) were lower in the Kidney-Heart service cohort versus the comparator cohort, although mean lengths of stay were longer (14 versus 11 days, *P* < 0.001.) Follow-up in nephrology clinic within the University of Washington system was low for AKI patients at 1.7%.

**Conclusions:**

Our single-center experience demonstrates a model by which a specialized cardio-nephrology service can be implemented and provide care for complex patients. Further implementation clinical trials are needed to determine whether integrated care models can improve cardiovascular-kidney-metabolic syndrome outcomes.

## Visual Abstract

**Figure:**
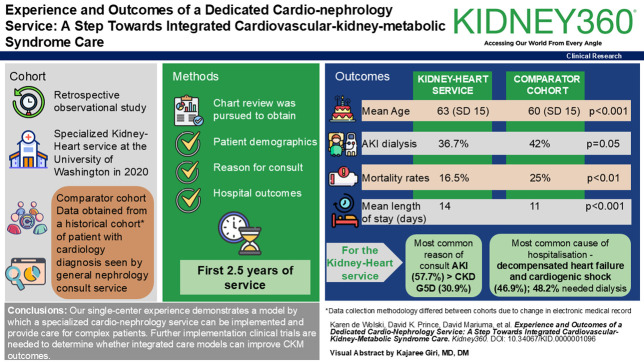

## Introduction

The American Heart Association recently proposed a new disease framework, the cardiovascular-kidney-metabolic syndrome^1^ (CKM), which affects up to 90% of US adults.^2,3^ The CKM is a multisystem disorder defined by interactions among different metabolic risk factors and kidney disease that increases the risk of cardiovascular disease. Various underlying pathophysiologic disruptions within these different organ systems have multidirectional consequences resulting in overall progression of CKM syndrome. Within this syndrome, the population of patients hospitalized with concomitant cardiac and kidney disease continues to grow and is associated with high morbidity and mortality compared with risk associated with either disease alone.^4–6^ Management of these patients is increasingly complex with modern advances in cardiac and nephrology care.^7,8^ As such, subspecialized cardio-nephrology care models may provide an opportunity to improve clinical outcomes in this patient population. As part of its presidential advisory, the American Heart Association had an urgent call to implement integrated care models to address the epidemic of CKM.^1^

To address the multidisciplinary challenges posed by this patient cohort, the Kidney-Heart service was launched in 2020 at the University of Washington Medical Center (UWMC).^9^ UWMC is a large quaternary care hospital that serves a broad cross-section of patients requiring advanced cardiac care, including heart transplants, complex percutaneous coronary intervention, and mechanical circulatory support (MCS) devices. The Kidney-Heart service is a consultation service comprised of nephrologists with specific cardiac expertise, and it covers all in-patient nephrology consults for patients on primary cardiac or cardiac surgery teams. These consults include primarily questions of AKI, dialysis-dependent CKD (CKD G5D), diuretic management, and electrolyte abnormalities.

We previously described the vision and mission of the UWMC Kidney-Heart service at the launch of this new service.^9^ The purpose of this descriptive observational study was to characterize the patients and the clinical outcomes observed on the Kidney-Heart service since its initiation. To assess whether there were any differences in these variables since starting the service, these data were compared with comparable data from cardiology patients requiring nephrology consults before the launch of the Kidney-Heart service.

## Methods

### Study Setting

This was a study of patients hospitalized at UWMC, a quaternary care hospital in Seattle, Washington, for primarily cardiac or cardiothoracic surgery indications.

### Kidney-Heart Service Timeline and Structure

The Kidney-Heart service was started in 2020 at UWMC. The service consists of typically two to three nephrologists who serve as a primary nephrology consultant for rotating weeks, such that each nephrologist is usually on service for one week at a time. The service was initially attending-only, but nephrology (and occasionally cardiology) fellows started rotating with this service about 50% of the time after seven months of its implementation. All nephrology consults at UWMC from primary cardiology or cardiothoracic surgery teams are directed to the Kidney-Heart service rather than the general nephrology service. The attending nephrologists were specifically trained in diuretic management, point-of-care-ultrasound, and cardiac devices, and they meet on a quarterly basis to discuss care patterns and management strategies. They work closely with cardiology providers to implement changes in diuretic recommendations and AKI prevention, including in cardiothoracic surgical patients and MCS patients. A standardized diuretic escalation protocol was designed through this service to assist both cardiology and nephrology teams with diuretic management in decompensated heart failure (Supplemental Figure 1).

### Data Collection: Kidney-Heart Service Cohort

A search of the current UWMC electronic medical record (EMR) was completed to identify patient encounters patients seen by the Kidney-Heart service (April 2021–November 2023). This did not include the first 7 months of the service due to an EMR change that was made during this time that did not allow for detailed chart review. This search was completed by searching for every patient encounter with a note written by one of the Kidney-Heart attending physicians during this period. Multiple encounters could be identified for the same patient. Additional demographic data, including age, sex, ethnicity, and insurance, were collected, along with information on the encounter such as intensive care unit (ICU) requirement, length of stay, 30-day readmission rate, mortality, creatinine patterns, CKD G5D status, and weight trends. Subsequently, a physician chart review was completed of each encounter to identify primary reasons for hospitalization, primary reasons for consult, dialysis, and MCS use. Multiple reasons for hospitalization and nephrology consultation could be identified for the same encounter.

### Historical Comparison Cohort Description and Data Collection

A search was completed of the historic EMR to identify patients who were on a primary cardiology or cardiothoracic surgery service with nephrology consults between January 2016 and December 2019. This was a different, now inactive EMR due to an institutional EMR change in 2020, which limited the ability to collect the same level of individualized patient data as the contemporary Kidney-Heart service cohort. This search was completed using team identification codes identified for each relevant service. Data collected included demographics, mortality, length of stay, ICU requirement, and 30-day readmission rate. CKD G5D, AKI, and inpatient dialysis use were obtained using International Classification of Diseases, Tenth Revision (ICD-10) codes.

### Analytic Approach

Statistical analyses were performed to characterize the distribution of patients across demographics, consult categories, and primary reasons for hospitalization. Statistical significance was calculated using the chi-squared test for dichotomous variables and *t* tests for continuous variables. Proportions were calculated to determine the relative representation of patients within each consult category, stratified by key clinical outcomes, including dialysis, ICU admission, in-hospital mortality, and 30 day readmission rates. Means or medians of continuous variables such as length of stay, change in weight over hospitalization, and creatinine values (Kidney-Heart service only) were calculated, stratified by consult category. The proportions of patients using various types of MCS and the percentage of those patients requiring dialysis within the Kidney-Heart service cohort were quantified. Analyses were performed in *R* version 4.4.3 (*R* Core Team, 2025).

## Results

### Characteristics of Patients Cared for on the Kidney-Heart Service and the General Nephrology Consult Service

The Kidney-Heart service was consulted for a total of 509 unique cardiac and cardiothoracic surgery patients with 605 encounters over 31 months. The mean number of encounters for each patient was 1.2 (SD of 0.7). The general nephrology consult service was consulted on a total of 720 unique cardiac and cardiothoracic surgery patients with 893 encounters over 48 months. Both services saw patient cohorts that were older in age and racially diverse, although patients seen by the Kidney-Heart service were older and were more likely to be male compared with the historical cohort. Patient insurance coverage trends were grossly similar with majority Medicare; however, a greater proportion of Medicare patients was seen by the Kidney-Heart service versus the historical cohort (Table 1).

**Table 1:** Characteristics of patients seen by the Kidney-Heart service (April 2021 to November 2023) compared with the general nephrology service (January 2016 to December 2019)

Patient Characteristics	Kidney-Heart Service	General Nephrology Service	Difference	*P* Value
**Demographics**				
Age (mean, yr)	62.9 (SD 15.1)	60 (SD 15)	2.9	<0.001
Male sex (%)	62.7	66	−3.6	<0.001
**Race**				
Asian American/Pacific Islander %	13.4	11.3	2.1	
Black %	12.2	8.9	3.3	
Native American %	1.8	1.5	0.3	
Unknown%	5.7	5.1	0.5	
White %	67	73.2	−6.0	0.20
**Insurance**				
Medicare %	66	58	5.3	<0.001
Private/commercial %	16	28	−13.4	
Medicaid %	15	12	3.7	
Case rate %	3	2	1.1	

### Hospital Diagnoses and Reason for Consult among Patients Seen by the Kidney-Heart Service

Approximately half of patients had a primary reason for hospitalization of heart failure decompensation and/or cardiogenic shock, although other primary reasons for hospitalization were included such as valvular disorders and acute coronary syndrome among others (Table 2).

**Table 2:** Hospital diagnoses, encounter number, and reason for consult among patients seen by the Kidney-Heart service

Consult Question	% of Encounters
**Hospitalization Characteristics**	**Numerical Data**
CKD G5D	30.9
AKI	57.7
Diuretic management	8.9
Electrolyte abnormalities	7.8
Advanced CKD	5.3
Other	1.3
**Encounters per patient**	
Mean (SD)	1.2 (0.7)
Median (IQR)	1 (1–1)
Minimum–maximum	1–7
**Primary hospitalization reason(s)** ^a^	**% Of encounters**
Heart failure/cardiogenic shock/volume overload	46.9
Cardiac arrest	3.5
Kidney injury/kidney disease	6.0
Acute coronary syndrome or PCI need	15.7
MCS complication	2.1
Heart transplant/transplant complications	13.4
Infection	9.1
Arrythmia	6.0
Valvular disease	13.7
Aortic disease	2.1
Electrolyte disorder	1.0
Other	5.5
**Kidney function trends**	**(Median, IQR)**
Peak creatinine (mg/dl)	4.0 (3.0–5.2)
Nadir creatinine (mg/dl)	1.4 (1.0–2.0)
Discharge creatinine (mg/dl)	2.2 (1.5–3.1)
**Weight trend by consult category**	**Weight change (kg): Admit to discharge, median (IQR)**
CKD G5D	−0.6 (−3.7 to 1.4)
AKI	−3.9 (−10.4 to 0.5)
Diuretic management	−6.6 (−12.6 to −3.6)
Electrolyte abnormality	−4.2 (−9.7 to −1.0)
CKD	−3.5 (−7.6 to 0.2)
Other	−7.8 (−14.6 to −2.4)
**MCS patients**	
Type of MCS (single encounter can have multiple)	***n* (%)**
*Left or right ventricular assist device*	57 (41.0)
*Impella*	48 (34.5)
*Intra-aorta balloon pump*	43 (30.9)
*Extra corporeal membranous oxygenation*	31 (22.3)
*Dialysis requirement*	67 (48.2)

CKD G5D, dialysis-dependent CKD; IQR, interquartile range; MCS, mechanical circulatory support; PCI, percutaneous coronary intervention.

a Can exceed 100% because multiple primary reasons for hospitalization per encounter could be identified.

The Kidney-Heart service was usually consulted early in admission (median day of first note was hospital day 1). In both the Kidney-Heart service and general nephrology cohorts, approximately one third of consults were for CKD G5D (30.9% and 33%, respectively). Approximately two thirds of consults were for AKI in the general nephrology group (63%). An additional portion of consults for the Kidney-Heart service was identified for diuretic management (8.9%), electrolyte abnormalities (7.8%), and advanced CKD (5.3%; Table 2). For patients with diuretic resistance, a standard protocol was implemented by the Kidney-Heart service (Supplemental Figure 1). The use of MCS was common (22%) in Kidney-Heart service patients (with ventricular assist devices and impellas the most common types) and almost half of those patients required dialysis (Table 2).

### The Kidney-Heart and General Nephrology Services: In-Hospital Outcomes

Patients in all consult categories lost weight from the time of admit to discharge, with this weight change being most notable in the diuretic management and “other” consult categories, and least pronounced among CKD G5D patients (Table 2). Kidney-Heart service patients with AKI during the hospitalization had improvements in the serum creatinine by discharge but, on average, did not return to the nadir serum creatinine (Table 2). Only two (1.7%) non-CKD G5D patients had nephrology follow-up within 60 days in the University of Washington system.

Patient cohorts for both services had high rates of ICU requirement, mortality, readmission rates, and long length of stay in most consult categories. The patients seen by the Kidney-Heart service had longer median lengths of stay but lower mortality rates (Table 3). The rate of AKI requiring dialysis (AKI-D) was also lower in the Kidney-Heart service cohort than the general nephrology cohort (Table 3), and 7.2% of AKI-D patients still required dialysis at the time of discharge.

**Table 3:** ICU requirement and hospital outcomes

Patient Outcomes	Kidney-Heart Service	General Nephrology Service^a^	Difference	*P* Value
**ICU requirement**				
CKD G5D%	47	53	−6.0	0.03
AKI%	73.6	78	−4.4	0.06
Diuretic management%	55.6	N/A	—	—
Electrolyte abnormalities%	72.3	N/A	—	—
**Hospital outcomes**				
Length of stay (d, median, [IQR])	14 (6.0–25)	11 (5.0–23)	3	<0.001
30 d readmission rate %	25	21.7	3.3	0.17
Inpatient mortality%	16.5	25	8.5	<0.01
AKI requiring dialysis%	36.7	42	−5.3	0.05
AKI-D, on dialysis at discharge%	7.2	N/A	—	—

AKI-D, AKI requiring dialysis; CKD G5D, dialysis-dependent CKD; ICD, International Classification of Diseases-Tenth Revision; ICU, intensive care unit; IQR, interquartile range.

a Categories where data were not available due to collection methodology (ICD-10 codes) are marked as N/A.

## Discussion

Patients with cardiac and kidney comorbidities present a significant medical management challenge, a challenge often amplified in the hospital setting with frequent dynamic changes in patients' conditions. In an attempt to address these complexities, the Kidney-Heart service was created to provide nephrology care for patients hospitalized for primarily cardiac indications. This study aimed to provide descriptive data on the patients that were cared for by a subspecialized nephrology consult service, the Kidney-Heart service. This study suggests that the patient cohort and CKD G5D distribution of the Kidney-Heart service was largely similar to that of cardiology patients cared for by general nephrology in prior years, although the cohort was older. This group of patients is very complex, with high rates of ICU utilization, long median length of stay, and high risk of 30-day readmission. These early descriptive data may suggest that implementation of a cardio-nephrology service to care for this highly complex population is a feasible intervention. It is possible that we observed trends in improvement in in-hospital outcomes among patients cared for by the Kidney-Heart service, although many confounders necessarily exist within this observational study that prevent drawing any direct conclusions on causation. Importantly, the pre-post design of the study may be subject to bias, including differences in management, providers, available interventions, patient complexity, the coronavirus disease 2019 pandemic, and many innovations in both cardiac and nephrology care that have occurred in this time frame. If possible, future randomized controlled trials would help address these biases. Therefore, the findings of this descriptive study should be interpreted in the context of these important limitations.

In this study, we observed a descriptive trend of lower mortality rate for the patient cohort cared for by the Kidney-Heart service compared with the general nephrology cohort, despite an older age and growth in the cardiology services at UWMC which have brought in patients with more complex disease and interventional need. Although reasons for this decline in mortality rate are surely multifactorial and likely largely due to a myriad of advancements in care, it remains reassuring that the initiation of the Kidney-Heart service did not correlate with higher mortality, despite the complexity of this patient cohort. Although we observed lower rates of mortality, length of stay and 30-day readmission rate were higher among the patients cared for by the Kidney-Heart service. The reasons for these conflicting observations are unclear. It is possible that these trends may reflect longer survival (*e.g*., patients reaching advanced heart failure therapies and discharge) among the patient cohort. They also may reflect an increase in complexity of cardiac procedures through this time correlating to the growth of the Heart Institute at UWMC. It is also possible that other barriers to discharge were more pronounced during this period. Further larger multicenter studies that are able to address for confounding would be important to verify these findings.

Descriptive trends suggesting possible lower rates of need for dialysis in AKI patients in the Kidney-Heart service cohort versus the historical general nephrology cohort (36.7% versus 42%) were observed in our study. Although it cannot be said that the service was causative of this trend, enhanced interdisciplinary relationships and expertise may have contributed to avoiding dialysis more commonly in AKI patients with diuretic resistance. One of the goals in starting this service was to reduce the rate of dialysis for volume overload by fostering expectation of nephrology involvement in complex diuresis before a dialysis need. Diuretic questions constituted almost 9% of all consults, demonstrating willingness to request nephrology expertise for this management. The Kidney-Heart service developed and implemented a standard diuretic algorithm (Supplemental Figure 1), which helped identify appropriate initial diuretic doses, used objective measures (*e.g*., urine sodium) for dose titration, and used multiple classes of diuretics to achieve sequential nephron blockade. It is possible that this approach contributed to greater diuretic responsiveness with reduced need for acute dialysis. Of note, the diuretic management consult category had the highest weight loss trend of any of the classified consult categories, although it must be recognized that many reasons, including nonfluid weight loss, could have accounted for this. It is also encouraging that only a small minority of AKI-D patients who survived to discharge had ongoing dialysis need, which may suggest close attention to renal recovery through the hospital course.

We also observed that follow-up of non-CKD G5D nephrology consult patients was quite low, although this may have been undercaptured due to follow-up care that could have occurred outside of the UW system. Nephrology follow-up of AKI patients is a known care-gap for which a number of barriers to appropriate care have been identified.^10,11^ This represents a potentially significant area for improvement given that these patients can be at high risk for CKD progression.^12,13^ Nephrology follow-up can allow for ongoing kidney risk stratification and reinitiation/titration of nephro-protective medications such as renin-angiotensin receptor blockade, which has been associated with improved mortality in many of these patients.^14^ In the last year, a multidisciplinary Kidney-Heart clinic was launched at UWMC in which patients with both cardiac and nephrology needs can be seen in concurrent consult with both a cardiologist and nephrologist. There are not yet data for this clinic, but we are hopeful that this may help address some of this need.

In the context of increasing health care costs, subspecialty services, such as the Kidney-Heart service, may offer economic advantages. The economic burden of kidney disease and cardiovascular disease is very high and is expected to increase in the next five years,^15^ with need for dialysis contributing to approximately 40% of that cost. The US Center for Medicare and Medicaid Services estimates for cost for an acute heart failure hospitalization are nearly $18,000, for a kidney related death $76,000, and for a cardiovascular death $30,000.^16,17^ Each hospital day for a heart failure admission is estimated at $100 to $2900.^18^ In the United States, AKI associated with percutaneous coronary intervention has been shown to increase the index admission costs by approximately $11,313 and total 1-year costs by $14,800. Similarly, AKI following major elective noncardiac surgery can increase the mean total costs of hospitalization to $33,042 for stage 1 AKI, $39,133 for stage 2 AKI, and $73,216 for stage 3 AKI, compared with $23,896 for patients without AKI.^19^ In those patients who require dialysis, the cost per session can be greater than $1000. It is therefore possible that a service associated with reduced need for acute dialysis could lead to overall cost-savings. However, this would need to be balanced with longer lengths of stay. Formal economic impact analyses are needed to truly quantify the economic effect of a cardio-nephrology inpatient service.

This study had several strengths. We were able to collect a broad array of data for these patients, and this was bolstered through physician chart review to more thoroughly describe the cohort. We tried to isolate a retrospective patient cohort that would be best matched to compare outcomes with the implementation of the Kidney-Heart service. There were notable limitations, including that ICD-10 codes had to be relied upon more heavily in the retrospective group given the EMR transition. We were also unable to obtain the same level of individual patient data, including comorbidities, to optimally control for confounding. This greatly limited the comparability of the cohorts, especially as important elements such as primary reason for hospitalization were not reliably comparable. It also limited our ability to detect reason for consult beyond AKI and CKD G5D in the retrospective cohort, which was based on ICD-10 codes instead of chart review. Therefore, consult questions, such as diuretic resistance and electrolyte abnormalities, which were likely present in the retrospective cohort, could not be detected. It is thus harder to determine whether there was a change in reason for consult over time with the implementation of Kidney-Heart service.

This was a descriptive study, and we had insufficient sample size to perform propensity score matching or adjust for all possible confounders. Notably, there have been significant advancements in cardiovascular and nephrology care during this period, including pharmacologic management strategies such as sodium-glucose cotransporter 2 inhibitor, and advancements in MCS devices and ICU care writ large, which have greatly changed patient outcomes.^20^ Importantly, the coronavirus disease 2019 pandemic coincided very closely with the implementation of this service. The consequences of the pandemic, which led to countless potential confounders, could not be well accounted for in this analysis, despite almost certain impacts on outcomes. Some potential considerations include that pandemic-related constraints on skilled nursing facility capacity may have led to longer lengths of stay, and suspended elective procedures may have led to both fewer short postprocedural hospitalizations and more delayed presentations to care. Any changes in outcomes patterns during this period must therefore be interpreted within the ambiguity of potential pandemic impacts. In addition, in this observational study, we are not able to determine whether there was a causal effect of the care provided by the Kidney-Heart service and the clinical course. There are of course countless other changes and advancements in care affecting patient outcomes that cannot be identified with a retrospective study. We hope that this study prompts future clinical trials to more adequately address bias from observational studies. This could include trials that include cardiology patients not requiring nephrology care to help control for temporal changes in cardiology care that may confound outcomes.

In conclusion, these early descriptive data support the feasibility of an interdisciplinary service such as the Kidney-Heart service to care for complex patients with CKM. Further studies, including clinical trials, are needed to determine whether such care models can improve patient CKM outcomes.

## Supplementary Material

## Data Availability

Original data generated for the study will be made available upon reasonable request to the corresponding author. Data Type: Raw Data/Source Data; Observational Data. Reason for Restricted Access: Original data includes deidentified individual patient data. This was obtained as a QI project with an IRB exemption. IRB approval would be required for release.

## Declarative Statements

This study is exempt from the Institutional Review Board or Ethics Committee approval, and I have provided the reason for exemption. This was a quality improvement study. The IRB exempted it from approval.

## Supplemental Material

Supplemental Figure 1. University of Washington Heart Failure Diuretic Protocol.
