# Supplementary material for: Experience and Outcomes of a Dedicated Cardio-Nephrology Service: A Step toward Integrated Cardiovascular-Kidney-Metabolic Syndrome Care
Source: Kidney360. 2025 Dec 24;7(4):816–22. doi: 10.34067/KID.0000001096 (PMC13134808; doi:10.34067/KID.0000001096)
Supplement: Supplementary file 1 [file kidney360-7-816-s001.pdf]

ASN American Society of Nephrology | JASN Journal of the American Society of Nephrology | KIDNEY360 Accessing Our World From Every Angle | CJASN Clinical Journal of the American Society of Nephrology

ASN Journal Disclosure Form

As per ASN journal policy, I have disclosed any financial relationships or commitments I have held in the past 36 months as included below. I have listed my Current Employer below to indicate there is a relationship requiring disclosure. If no relationship exists, my Current Employer is not listed.

N. Arora reports the following:
Consultancy: Novo Nordisk; Research Funding: Novo Nordisk; Eli Lilly; and Speakers Bureau: Bayer Pharmaceuticals.

I understand that the information above will be published within the journal article, if accepted, and that failure to comply and/or to accurately and completely report the potential financial conflicts of interest could lead to the following: 1) Prior to publication, article rejection, or 2) Post-publication, sanctions ranging from, but not limited to, issuing a correction, reporting the inaccurate information to the authors' institution, banning authors from submitting work to ASN journals for varying lengths of time, and/or retraction of the published work.

Name: Nayan Arora
Manuscript ID: K360-2025-000997R1
Manuscript Title: Experience and outcomes of a dedicated cardio-nephrology service: a step towards integrated cardiovascular-kidney-metabolic syndrome care
Date of Completion: November 3, 2025
Disclosure Updated Date: July 22, 2025

ASN American Society of Nephrology | JASN Journal of the American Society of Nephrology | KIDNEY360 Accessing Our World From Every Angle | CJASN Clinical Journal of the American Society of Nephrology

ASN Journal Disclosure Form

As per ASN journal policy, I have disclosed any financial relationships or commitments I have held in the past 36 months as included below. I have listed my Current Employer below to indicate there is a relationship requiring disclosure. If no relationship exists, my Current Employer is not listed.

N. Bansal reports the following:
Employer: University of Washington; Consultancy: AstraZeneca; Patents or Royalties: UptoDate; and Advisory or Leadership Role: Kidney360 Associate Editor; American College of Physicians.

I understand that the information above will be published within the journal article, if accepted, and that failure to comply and/or to accurately and completely report the potential financial conflicts of interest could lead to the following: 1) Prior to publication, article rejection, or 2) Post-publication, sanctions ranging from, but not limited to, issuing a correction, reporting the inaccurate information to the authors' institution, banning authors from submitting work to ASN journals for varying lengths of time, and/or retraction of the published work.

Name: Nisha Bansal
Manuscript ID: K360-2025-000997R1
Manuscript Title: Experience and outcomes of a dedicated cardio-nephrology service: a step towards integrated cardiovascular-kidney-metabolic syndrome care
Date of Completion: October 20, 2025
Disclosure Updated Date: January 7, 2025

ASN American Society of Nephrology | JASN Journal of the American Society of Nephrology | KIDNEY360 Accessing Our World From Every Angle | CJASN Clinical Journal of the American Society of Nephrology

ASN Journal Disclosure Form

As per ASN journal policy, I have disclosed any financial relationships or commitments I have held in the past 36 months as included below. I have listed my Current Employer below to indicate there is a relationship requiring disclosure. If no relationship exists, my Current Employer is not listed.

K. de Wolski reports the following:
Employer: University of Washington; Spouse: Mawer Investment Management; and Research Funding: Site PI for AstraZeneca clinical trial.

I understand that the information above will be published within the journal article, if accepted, and that failure to comply and/or to accurately and completely report the potential financial conflicts of interest could lead to the following: 1) Prior to publication, article rejection, or 2) Post-publication, sanctions ranging from, but not limited to, issuing a correction, reporting the inaccurate information to the authors' institution, banning authors from submitting work to ASN journals for varying lengths of time, and/or retraction of the published work.

Name: Karen S. de Wolski
Manuscript ID: K360-2025-000997R1
Manuscript Title: Experience and outcomes of a dedicated cardio-nephrology service: a step towards integrated cardiovascular-kidney-metabolic syndrome care
Date of Completion: October 20, 2025
Disclosure Updated Date: September 18, 2025

ASN
American Society of Nephrology
JASN
Journal of the American Society of Nephrology
KIDNEY360
Accessing Our World From Every Angle
CJASN
Clinical Journal of the American Society of Nephrology

ASN Journal Disclosure Form

As per ASN journal policy, I have disclosed any financial relationships or commitments I have held in the past 36 months as included below. I have listed my Current Employer below to indicate there is a relationship requiring disclosure. If no relationship exists, my Current Employer is not listed.

A. Gupta reports the following:
Employer: University of Washington

I understand that the information above will be published within the journal article, if accepted, and that failure to comply and/or to accurately and completely report the potential financial conflicts of interest could lead to the following: 1) Prior to publication, article rejection, or 2) Post-publication, sanctions ranging from, but not limited to, issuing a correction, reporting the inaccurate information to the authors' institution, banning authors from submitting work to ASN journals for varying lengths of time, and/or retraction of the published work.

Name: Ayushi Gupta
Manuscript ID: K360-2025-000997R1
Manuscript Title: Experience and outcomes of a dedicated cardio-nephrology service: a step towards integrated cardiovascular-kidney-metabolic syndrome care
Date of Completion: October 27, 2025
Disclosure Updated Date: October 27, 2025

ASN
American Society of Nephrology
JASN
Journal of the American Society of Nephrology
KIDNEY360
Accessing Our World From Every Angle
CJASN
Clinical Journal of the American Society of Nephrology

ASN Journal Disclosure Form

As per ASN journal policy, I have disclosed any financial relationships or commitments I have held in the past 36 months as included below. I have listed my Current Employer below to indicate there is a relationship requiring disclosure. If no relationship exists, my Current Employer is not listed.

D. Mariuma reports the following:
Employer: Amazon; and Ownership Interest: Amazon.

I understand that the information above will be published within the journal article, if accepted, and that failure to comply and/or to accurately and completely report the potential financial conflicts of interest could lead to the following: 1) Prior to publication, article rejection, or 2) Post-publication, sanctions ranging from, but not limited to, issuing a correction, reporting the inaccurate information to the authors' institution, banning authors from submitting work to ASN journals for varying lengths of time, and/or retraction of the published work.

Name: David Mariuma
Manuscript ID: K360-2025-000997R1
Manuscript Title: Experience and outcomes of a dedicated cardio-nephrology service: a step towards integrated cardiovascular-kidney-metabolic syndrome care
Date of Completion: October 20, 2025
Disclosure Updated Date: October 20, 2025

ASN American Society of Nephrology · JASN Journal of the American Society of Nephrology · KIDNEY360 Accessing Our World From Every Angle · CJASN Clinical Journal of the American Society of Nephrology

ASN Journal Disclosure Form

As per ASN journal policy, I have disclosed any financial relationships or commitments I have held in the past 36 months as included below. I have listed my Current Employer below to indicate there is a relationship requiring disclosure. If no relationship exists, my Current Employer is not listed.

D. Prince reports the following:
Employer: University of Washington

I understand that the information above will be published within the journal article, if accepted, and that failure to comply and/or to accurately and completely report the potential financial conflicts of interest could lead to the following: 1) Prior to publication, article rejection, or 2) Post-publication, sanctions ranging from, but not limited to, issuing a correction, reporting the inaccurate information to the authors' institution, banning authors from submitting work to ASN journals for varying lengths of time, and/or retraction of the published work.

Name: David K. Prince
Manuscript ID: K360-2025-000997R1
Manuscript Title: Experience and outcomes of a dedicated cardio-nephrology service: a step towards integrated cardiovascular-kidney-metabolic syndrome care
Date of Completion: October 20, 2025
Disclosure Updated Date: October 20, 2025

ASN American Society of Nephrology | JASN Journal of the American Society of Nephrology | KIDNEY360 Accessing Our World From Every Angle | CJASN Clinical Journal of the American Society of Nephrology

ASN Journal Disclosure Form

As per ASN journal policy, I have disclosed any financial relationships or commitments I have held in the past 36 months as included below. I have listed my Current Employer below to indicate there is a relationship requiring disclosure. If no relationship exists, my Current Employer is not listed.

T. Velu has nothing to disclose.

I understand that the information above will be published within the journal article, if accepted, and that failure to comply and/or to accurately and completely report the potential financial conflicts of interest could lead to the following: 1) Prior to publication, article rejection, or 2) Post-publication, sanctions ranging from, but not limited to, issuing a correction, reporting the inaccurate information to the authors' institution, banning authors from submitting work to ASN journals for varying lengths of time, and/or retraction of the published work.

Name: Tejas N Velu
Manuscript ID: K360-2025-000997R1
Manuscript Title: Experience and outcomes of a dedicated cardio-nephrology service: a step towards integrated cardiovascular-kidney-metabolic syndrome care
Date of Completion: October 20, 2025
Disclosure Updated Date: October 20, 2025
