# Supplementary material for: Experience and Outcomes of a Dedicated Cardio-Nephrology Service: A Step toward Integrated Cardiovascular-Kidney-Metabolic Syndrome Care
Source: Kidney360. 2025 Dec 24;7(4):816–22. doi: 10.34067/KID.0000001096 (PMC13134808; doi:10.34067/KID.0000001096)
Supplement: Supplementary file 2 [file kidney360-7-816-s002.pdf]

<u>Step 1</u>. Initial Dose of total IV loop diuretic/day is 2.5x outpatient dose given as an initial bolus with a continuous drip. Suggested dosing is below. ***Start at HIGHER STEP if: eGFR<30 ml/min/1.73 m2 OR Serum albumin <2.5 mEq/dL***

Step	Daily Outpatient Dose* Furosemide Equivalents (Mg/Day)	IV Bolus/Infusion Dosing (Preferred)	IV Bolus (Alternative)	Consider adjunctive Therapy? (see STEP 3)
A	**0-40** mg	**20** mg IV bolus + **2.5** mg/hr	**40** mg IV q**12**h	--
B	**41-80** mg	**40** mg IV bolus + **5** mg/hr)	**80** mg IV q**12**h	--
C	**81-120** mg	**80** mg IV bolus + **10** mg/hr	**160** mg IV q**12**h	**Yes**
D	**121-240** mg	**120** mg IV bolus + **15-20** mg/hr	**200** mg IV q**8**h	**Yes**
E	**>240** mg	**160** mg IV bolus + **20-30** mg /hr	**200** mg IV q**6**h	**Yes**
F	**Bumetanide**	**2-5 mg IV bolus + 0.5-2 mg/hr**	**1-3** mg IV q**12**h	**Yes**

* <u>Oral Diuretic Dosage Conversion</u>: 10 mg Torsemide = 20 mg Furosemide; 1 mg Bumetanide = 40 mg Furosemide

<u>STEP 2</u>. Diuretic Titration:

a. **First 2 hours and with any diuretic therapy change**: If Urine Na < 50-80 mmol/L, then INCREASE diuretic dose
b. **First 6-12 hours**: If Urine output <150ml/hour, then INCREASE diuretic dose
c. **Please check basic metabolic panel + magnesium twice daily and replete as necessary**
d. **Daily Assessment/Adjustment**: Check Weight Change/Spot Urine Sodium and Creatinine; ADJUST dose as follows:

Daily weight Change OR Spot Urine Sodium	<1 kg/ <50-80 mmol/L	1-3 kg/ ≥50-80 mmol/L	≥3 kg
Action	*INCREASE Diuretic Dose (per STEP 1) and/or ADD Adjunctive Therapy (see STEP 3)*	*Maintain*	*Decrease Step*
**Serum Creatinine**	**>30-50% Increase**		
Action	*Consider reassessment of volume status, and/or Nephrology consultation*		

e. **Failure to respond in 48-72 hours, go to STEP 3 and consider hemodynamic assessments**

<u>STEP 3</u>. Adjunctive therapies (**Consider Nephrology consultation and hemodynamic assessment if no response**):
*No evidence for low dose dopamine, or vasopressin antagonists to increase natriuresis

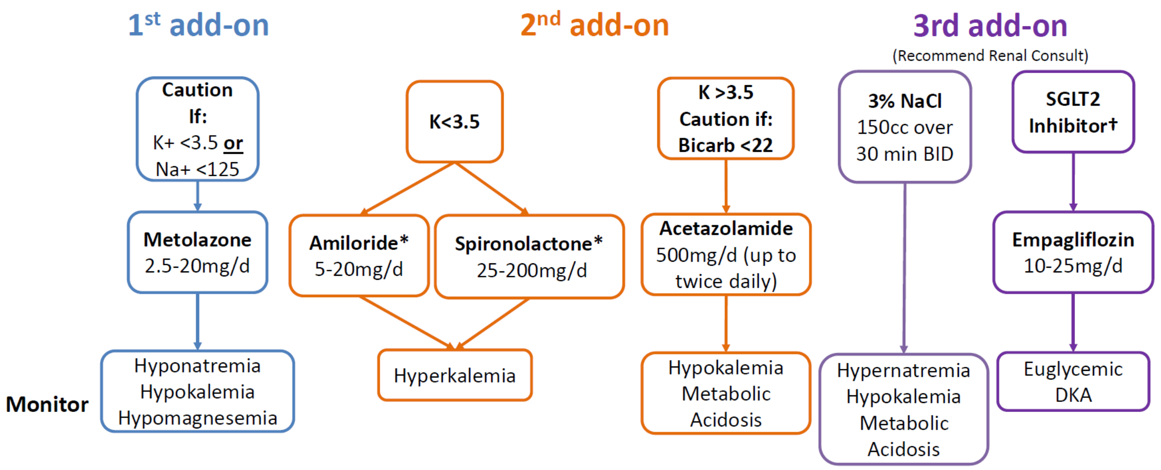

*Amiloride vs. Spironolactone: Spironolactone may require higher dose and take longer to achieve effect
† Guideline directed medical therapy; Empagliflozin 25mg may be needed for a diuretic effect

<u>STEP 4</u>. When "euvolemic":

- Use **Table 1** to convert Protocol Dose back to Home Dose and adust electrolyte monitoring/supplementation.
- Clues to **"clinical euvolemia"** include:
  - **a) At previous "dry" weight** (*ideally based on Right Heart Cath*), **b) JVP** and **c)** at **"baseline" BNP** level.
- **Confirm that <u>Weight</u> has <u>Decreased</u>!**
